# Radiotherapy for Merkel cell carcinoma: recommendations from the DEGRO Dermatooncology Working Group

**DOI:** 10.1007/s00066-026-02587-0

**Published:** 2026-09-09

**Authors:** Khaled Elsayad, Isabel Falke, Maike Trommer, Tina Jost, Alexander Rühle, Ahmed Gawish, Siyer Roohani, André Glowka, Markus Hecht, Eleni Gkika, Kathrin Hering, Laila König, Stefanie Corradini, Hans Theodor Eich, Dirk Vordermark

**Affiliations:** 1 Philipps-Universität Marburg, Marburg, Germany; 2 Klinik für Strahlentherapie, Universitätsklinikum Gießen – Marburg, Marburg, Germany; 3 Marburg Ion-Beam Therapy Center (MIT), Department of Radiotherapy and Radiation Oncology, Marburg University Hospital, Philipps-University Marburg, Marburg, Germany; 4 University Cancer Center (UCT) Frankfurt-Marburg, Marburg, Germany; 5 Klinik für Strahlentherapie, Universitätsklinikum Bonn, Bonn, Germany; 6 Klinik für Strahlentherapie, Ludgerus-Kliniken, Clemenshospital, Münster, Germany; 7 Universitätsklinikum Erlangen, Strahlenklinik, Friedrich-Alexander-Universität Erlangen-Nürnberg, Erlangen, Germany; 8 Klinik und Poliklinik für Strahlentherapie, Universitätsklinikum Leipzig, Leipzig, Germany; 9 Department of Radiotherapy and Radiation Oncology, University Medical Center Hamburg-Eppendorf, Hamburg, Germany; 10 Department of Radiation Oncology, University Hospital Magdeburg A. ö. R, Magdeburg, Germany; 11 Department of Radiation Oncology, Saarland University Medical Center, 66421 Homburg, Saar, Germany; 12 Klinik für Radioonkologie und Strahlentherapie, Universitätsklinikum Heidelberg, Heidelberg, Germany; 13 Klinik für Strahlentherapie – Radioonkologie, Universitätsklinikum Münster, Münster, Germany; 14 Universitätsklinik und Poliklinik für Strahlentherapie, Universitätsmedizin Halle, Halle, Germany

**Keywords:** Definitive, Curative, Palliative, Dose deescalation, Skin tumor, Hypofractionated, Ultrahypofractionated, Immunotherapy

## Abstract

Merkel cell carcinoma (MCC) is a rare malignancy with an annual incidence in Germany of 5.2 per million for men and 3.8 per million for women, predominantly affecting sun-exposed areas in older adults. Despite a frequently unfavorable prognosis, outcomes are multifactorial and depend on tumor site, disease stage, performance status, patient age, sex, and immune competence. The current standard of care prescribes surgical resection followed by adjuvant radiotherapy of the tumor bed, ideally initiated within 8 weeks after surgery to optimize clinical outcomes. In case of lymph node involvement, lymph node dissection and/or radiotherapy of the involved nodal basin are recommended. Furthermore, new evidence supports the effectiveness of shorter radiotherapy approaches, with moderate hypofractionated or even ultrahypofractionated schedules serving as effective options for certain patients, particularly those with frailty or comorbidities, or when it is clinically necessary to minimize the treatment burden. In cases of inoperability, refusal of surgery, or significant frailty, definitive local radiotherapy can achieve sufficient disease control. The synergistic potential of combining immunotherapy with radiation in advanced cases remains a key area for ongoing research.

## Introduction

Merkel cell carcinoma (MCC) is an infrequent but highly aggressive neuroendocrine malignancy of the skin. Its global incidence is rising significantly, a trend linked to population aging and cumulative ultraviolet (UV) radiation [1–5]. In Germany, the disease mainly affects older adults (median age 74), with incidence rates of 5.2 and 3.8 per million person-years in men and women, respectively [6]. The molecular pathogenesis is characterized by either Merkel cell polyomavirus (MCPyV) integration or UV-mediated mutational signatures. Often presenting as a rapidly expanding, asymptomatic, violaceous nodule, typically on the head, neck, or extremities, MCC carries a high risk for locoregional recurrence and systemic spread [7, 8].

Prognosis is primarily determined by disease staging but is also influenced by immune competence and histopathological markers such as lymphovascular invasion (LVI) and tumor depth. Overall survival (OS) at 5 years for MCC patients varies between 48% and 63%, mainly dominated by stage: 64% for stages I–II (American Joint Committee on Cancer [AJCC] 8th edition), 51% for node-positive stage III, and 68% for cases with an unknown primary. For stage IV metastatic disease, 5‑year OS remains poor, ranging from 17% to 29% [7, 8]. The current therapeutic gold standard for localized and regional disease (stages I–III) remains wide local excision (WLE) followed by adjuvant radiotherapy (RT) [1, 2, 9, 10].

**Fig. 1:**
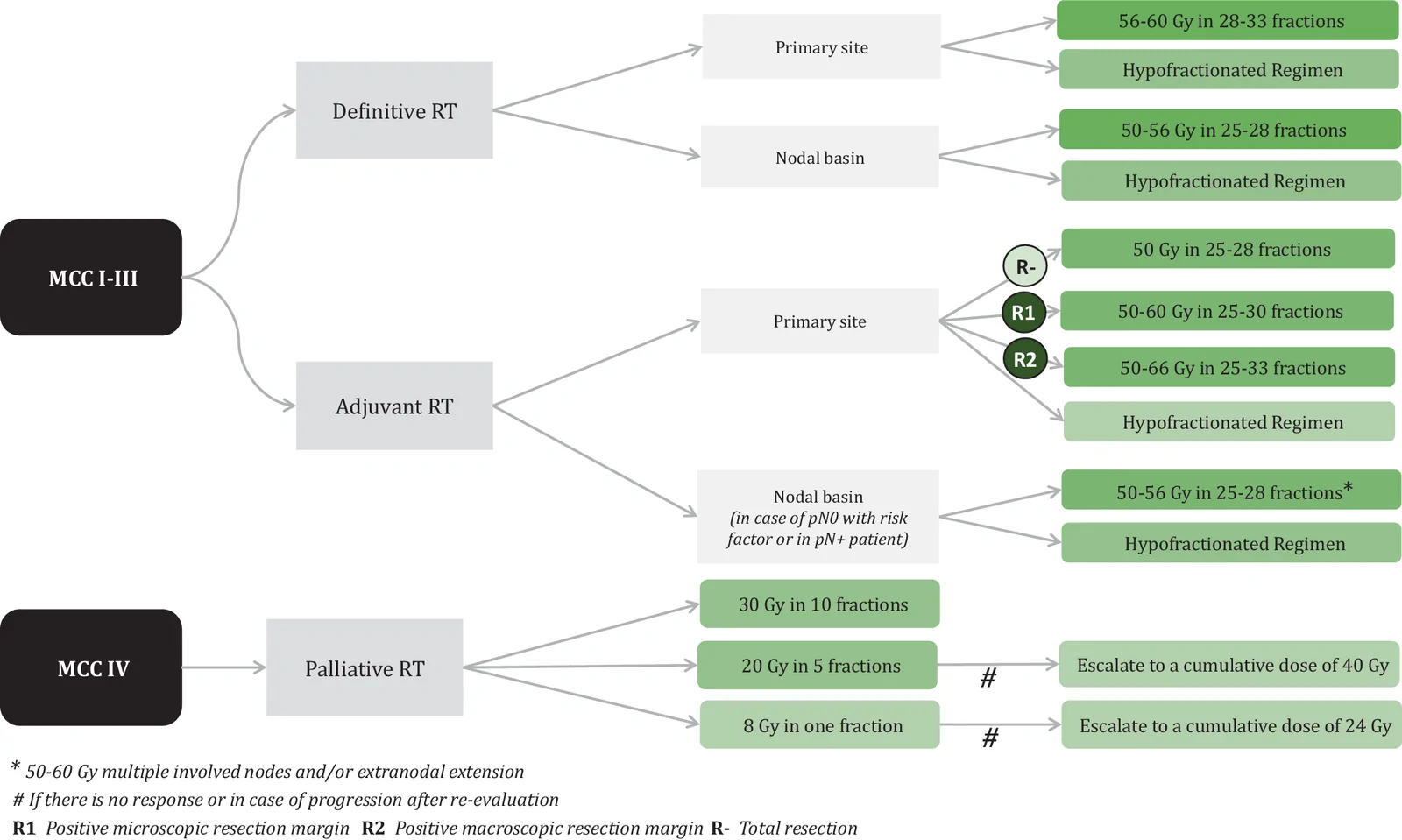
Overview of radiotherapy (*RT*) dose options for Merkel cell carcinoma (*MCC*)

While the German S2k guidelines generally focus on conventionally fractionated radiotherapy protocols and mention hypofractionation primarily in palliative situations, international guidelines have incorporated general recommendations for hypofractionation [2, 3, 11, 12]. Integration of these regimens reflects current clinical data demonstrating that shortened radiation courses achieve comparable efficacy to conventional fractionation. Both moderate and ultrahypofractionation have proven effective for specific cohorts, allowing for more tailored treatment strategies. In patients with inoperable disease, significant frailty, or substantial comorbidities, definitive local RT alone can yield acceptable local control [45, 46]. Furthermore, the synergistic potential of integrating immunotherapy with radiation in advanced MCC warrants continued investigation. This expert opinion from the German Society for Radiation Oncology (DEGRO) Dermatooncology Working Group evaluates radiotherapy and diverse fractionation schedules for both adjuvant and definitive settings, offering evidence-based dose recommendations for MCC management (Tables 1, 2, 3 and Fig. 1). These recommendations are based on a narrative review of current international guidelines and available clinical evidence, followed by expert consensus within the DEGRO Dermatooncology Working Group.

**Table 1:** Primary definitive hypofractionated radiotherapy regimens of Merkel cell carcinoma

*Primary definitive radiotherapy for primary disease*	
Hypofractionated regimens	2.25–2.5 Gy × 20 fractions
	3 G y × 10–15 fractions
	4 Gy × 10 fractions
	6 Gy × 5–6 fractions (non-consecutive days)
	8 Gy × 3 fractions (non-consecutive days)
*Primary definitive radiotherapy for nodal disease*	
Hypofractionated regimens	2.25–2.5 Gy × 20 fractions
	3 Gy × 10–15 fractions
	4 Gy × 10 fractions
	6 Gy × 5–6 fractions (non-consecutive days)
	8 Gy × 3 fractions (non-consecutive days)

**Table 2:** Postoperative hypofractionated radiotherapy regimens for Merkel cell carcinoma (within 8 weeks after surgery)

*Radiotherapy to the primary site*	
Hypofractionated regimens	2.25–2.5 Gy × 20 fractions
	3–4 Gy × 10 fractions
	8 Gy × 1 fraction (in selected low-risk patients)
*Radiotherapy to the regional nodal basin*	
Hypofractionated regimens	2.25–2.5 Gy × 20 fractions
	3–4 Gy × 10 fractions
	8 Gy × 1 fraction (in selected low-risk patients)

**Table 3:** Palliative radiotherapy regimens for Merkel cell carcinoma (sympotomatic or progressive lesions)

Hypofractionation	3 Gy × 10 fractions
	4 Gy × 5 fractions, with the possibility of repetition
	8 Gy × 1 fractions, with the possibility of repetition

## Radiobiological background

The pathogenesis and radiobiologic behavior of MCC are fundamentally driven by two primary risk factors: chronic UV radiation exposure and Merkel cell polyomavirus (MCPyV) infection [13]. While MCPyV is ubiquitously present on the skin of most healthy individuals, viral sequences are integrated into the host genome in approximately 80% of tumors [13]. These virus-associated MCCs are typically characterized by a low tumor mutational burden (TMB). In contrast, UV-associated MCCs exhibit a high TMB, which often includes mutations in critical tumor suppressors like p53 and pRB as well as various DNA-damage repair (DDR) proteins. Analysis by Dai et al. showed a negative correlation of high TMB and radiosensitivity [14]. In contrast, a high TMB is also discussed as potential biomarker for a positive response to immune checkpoint blockade [15]. General immunogenicity is a hallmark of MCC, as evidenced by the significantly increased incidence in immunosuppressed and human immunodeficiency virus (HIV) patients, as well as observed instances of spontaneous tumor regression, which is observed in rare case. However, the tumor often employs complex immune escape mechanisms to bypass these responses [3]. From a radiobiologic perspective, the role of DDR pathways is interesting but also critical; research indicates that MCPyV-positive MCCs may over-express DDR genes such as *ATM* and *ATR* as well as their downstream kinases *Chk1* and *Chk2*, suggesting viral interference with cellular repair mechanisms [16]. However, mutations in genes of central DDR proteins are known to intensify the effects of DNA-damaging RT. Further, investigational strategies are currently exploring the use of Janus kinase (JAK) inhibitors as potential radiosensitizers [17]—a concept currently under study in head and neck squamous cell carcinoma (HNSCC; NCT07457346)—and agents like selinexor, which has been shown to downregulate DDR protein expression in both virus-positive and virus-negative MCC cell lines [18]. These molecular characteristics underscore the tumor’s high degree of radiosensitivity and the potential for synergistic combinations with modern systemic therapies [19].

## Recommendations for adjuvant radiotherapy in localized (stage I-II) and locoregionally advanced (stage III) MCC

### Primary tumor bed

Traditional management of localized MCC consists of surgical resection and nodal staging via sentinel lymph node biopsy (SLNB), typically followed by adjuvant RT. This multimodal approach markedly reduces recurrence rates and is linked to superior disease-specific survival (DSS) [13]. The surgery-to-radiotherapy interval is a critical prognostic determinant. Initiation of RT within 8 weeks after surgery, or within 79 days of diagnosis, is associated with significantly better outcome. Delays exceeding this window substantially elevate the risk of locoregional failure [11, 20–22].

Evidence from a large-scale systematic review of more than 17,000 patients supports the use of adjuvant RT after surgical resection for stage I–III MCC, proving significant improvement in OS as well as local and locoregional disease-free survival (DFS) [23]. Data from the National Cancer Database involving 6908 stage I–II MCC patients confirm that surgery followed by adjuvant RT significantly improves OS compared to monotherapy. Notably, this survival benefit did not extend to stage III patients, for whom neither adjuvant RT nor chemotherapy showed a statistically significant effect on OS [22].

An additional analysis of 2330 stage I–II MCC patients from the National Cancer Database underscores the impact of appropriate adjuvant therapy. Despite a 5-year OS benefit (76% with RT vs. 68% without RT), the study found that 43% of eligible patients were undertreated. Simultaneously, 43% of patients received RT despite not meeting standard criteria. These findings highlight that receiving RT when indicated significantly improves long-term survival [24]. Most patients are treated with doses between 50 and 65 Gy, as recommended in guidelines [2, 12, 13]. In terms of dose optimization, a retrospective analysis of 2093 patients with stage I–III MCC of the extremities or trunk evaluated various radiotherapy regimens (ranging from 30 to 70 Gy in conventional fractionations). The study concluded that adjuvant doses between 40 Gy and <50 Gy appear sufficient, as they yielded OS outcomes equivalent to those achieved with higher-dose regimens (> 50–70 Gy) [25]. A recent retrospective evaluation of 324 patients with 618 treated tumor sites found no significant association between radiation dose and clinical outcomes in terms of local and distant tumor control. Local tumor control (3-year local progression-free probability) was 94% for the entire cohort across the entire spectrum of delivered doses, ranging from low palliative doses (e.g., 1 × 8 Gy) to high definitive doses, supporting the consideration of dose de-escalation in selected patients [26].

### Regional nodal radiotherapy

#### Local MCC (stage I–II; N0)

While adjuvant RT is not routinely recommended for pN0 disease, it remains a consideration for high-risk patients (tumor size > 2 cm, immunosuppression, or LVI). Elective regional radiotherapy is indicated when SLNB is not feasible or its accuracy is compromised—such as in midline trunk or head and neck tumors with complex drainage basins—and in cases of severe immunosuppression. Occult metastasis is common in MCC, occurring in up to approximately 25% of patients [27, 28]. The only randomized prospective trial in MCC proved that adjuvant regional RT significantly decreased regional relapse rates in stage I MCC, with a 3-year regional recurrence rate of 0% vs. 17%. However, no survival benefit was demonstrated [29]. Significantly higher regional recurrence-free survival for patients receiving adjuvant regional RT of the lymph nodes after surgery of the primary tumor was shown in a cohort of 62 patients with early-stage MCC (84% vs. 43%). The authors also proved significantly higher 2‑year local recurrence-free survival after radiotherapy to the primary site (89% vs. 36%) [30].

#### Locoregional MCC (stage III; T1–4 N1–3 M0 disease)

For patients with a positive sentinel lymph node biopsy (pN1a), adjuvant radiotherapy to the involved nodal site is indicated as either an alternative or an adjunct to complete lymph node dissection (CLND). In cases of clinically positive nodes characterized by multiple involvements or extranodal extension, regional LN dissection followed by postoperative radiotherapy is recommended. Definitive radiotherapy remains the standard for inoperable candidates. Adjuvant chemotherapy is currently not recommended; instead, patients should be encouraged to participate in clinical trials investigating (neo)adjuvant systemic immunotherapy [12]. Comparative studies between CLND and radiotherapy [31], or CLND versus observation with or without adjuvant radiation, indicate that CLND alone is associated with inferior survival outcomes in SLNB-positive patients [32]. Definitive radiotherapy should be presented as a viable primary treatment option within a shared decision-making framework. This approach is particularly relevant for older patients, those with significant comorbidities, or in cases where surgical intervention would result in poor aesthetic outcomes and high functional morbidity due to challenging anatomical locations.

#### Advanced MCC (stage IV)

For patients with unresectable, recurrent, or metastatic MCC, immunotherapy is the established standard of care [12, 13]. However, the potential synergistic effects of combining immunotherapy with radiotherapy require further investigation. In a recent phase II trial involving 50 patients who received either first- or second-line nivolumab/ipilimumab, researchers evaluated the efficacy of concurrent stereotactic body radiation therapy (SBRT) delivered to at least one lesion (3 × 8 Gy; equivalent dose in 2‑Gy fractions [EQD2; α/β = 10 Gy]: 36 Gy). While the combination proved safe and achieved excellent local tumor control, the specific additive benefit of SBRT remains unproven. This is largely attributed to the study’s small cohort and the exceptional 100% response rate observed in the immunotherapy-only first-line group [33]. Single-fraction radiotherapy has shown excellent in-field control rates and durable responses at treated sites with minimal toxicity in a cohort of 26 patients with 93 MCC lesions. More precisely, the objective response rate among measurable irradiated tumors was 94%, with complete responses observed in 45% of lesions. Single-fraction radiotherapy provided complete pain resolution in 63% of patients with bone metastases and a marked relief in the remaining 37%. Interestingly, the authors found a significant link between the patient’s immune status and the long-term success of radiotherapy, with lower in-field progression rates in patients with no prior chemotherapy or known immunosuppression [34].

## Radiotherapy doses and fractionation regimens

Evidence from several small retrospective cohorts indicates no significant differences in local control or DSS when comparing hypofractionated and standard-course radiotherapy for stages I–III MCC [35, 36]. If a hypofractionated regimen is selected, current National Comprehensive Cancer Network (NCCN.org) guidelines recommend hypofractionated regimens such as 44–50 Gy delivered in 20 fractions (EQD2: ~45.8–52.1 Gy) or 30–35 Gy in 10 fractions (EQD2: ~32.5–39.4 Gy). For highly selected frail patients following R0 resection or those with minimal recurrence risk, a single-fraction dose of 1 × 8 Gy (EQD2: 12 Gy) may be considered [12, 37, 38]. In a comparative analysis of dosing schedules, a hypofractionated regimen of 3 × 8 Gy (EQD2: 36 Gy) demonstrated superior efficacy over a single-fraction 8‑Gy dose (EQD2: 12 Gy). In a cohort of 20 patients with 67 treated lesions, the multi-fraction approach yielded higher complete response rates and a significantly lower incidence of local recurrences [39]. A retrospective analysis of 24 patients (29 treatment courses) compared three radiotherapy regimens: standard fractionation in curative intent (EQD2: 50–60 Gy), hypofractionated radiotherapy in curative intent (10 × 4 Gy; EQD2: 46.67 Gy), and a single-fraction palliative dose (1 × 8 Gy; EQD2: 12 Gy). The results demonstrated that hypofractionated radiotherapy achieved clinical outcomes and toxicity profiles comparable to standard fractionation, while significantly reducing the overall treatment burden. An ongoing clinical study is currently recruiting to determine the disease outcome of patients receiving hypofractionated radiotherapy to the primary tumor site/bed and/or regional node basin (Clinicaltrials: NCT05100095).

## Target volume definition and radiotherapy technique

A clinical target volume (CTV) margin of 3–5 cm around the tumor bed is recommended [12, 13]. To ensure adequate dosing at the skin level, use of a bolus is recommended for the primary site. The selection between electron and photon beam therapy for MCC is primarily dictated by the target volume depth and anatomical complexity; while electron therapy remains an excellent choice for superficial, skin-confined lesions to minimize the integral dose to underlying structures, megavoltage photons (utilizing intensity-modulated radiation therapy [IMRT] or volumetric arc therapy [VMAT]) with image-guidance are the preferred modality for deep-seated infiltrations and the comprehensive management of regional lymph node basins.

## Molecular imaging

Fluorodeoxyglucose positron-emission tomography/computed tomography (FDG-PET/CT) in MCC is highly recommended due to its superior sensitivity for detecting occult metastatic disease and its subsequent impact on clinical staging. Furthermore, it facilitates biologically adapted radiotherapy planning and ensures precise assessment of the treatment response during immune checkpoint blockade. Imaging via PET/CT can lead to upstaging in up to 30%–40% of patients, thus necessitating a more intensified therapeutic strategy [40–42]. Furthermore, PET/CT scans for restaging can be used for further treatment modulation, as a predictor of survival in the case of complete metabolic response, or to help identify patients for salvage treatments after locoregional recurrence [43].

## Definitive radiotherapy

To date, there are no prospective trials directly comparing definitive radiotherapy (RT) to the standard approach of surgery followed by adjuvant RT for MCC. Surgery can be clinically challenging or contraindicated in older patients with significant comorbidities, particularly for lesions in the head and neck region, where functional or cosmetic preservation is a priority. Small retrospective analyses have demonstrated no statistically significant differences in OS or DFS for stage I MCC when comparing definitive RT to surgery plus adjuvant RT [44–46]. Specifically, research indicates that higher-dose definitive RT (median 65 Gy) can achieve excellent local control; in some cohorts, it has even shown lower locoregional relapse rates (8% vs. 16%) compared to surgery plus adjuvant RT (median 55 Gy) [38]. Notably, the definitive RT group, receiving a median dose of 60 Gy, demonstrated a lower local recurrence rate (14%) than the cohort undergoing surgery followed by adjuvant radiotherapy to 50 Gy, which showed a recurrence rate of 26% [47]. Definitive radiotherapy (RT) offers superior aesthetic and functional preservation compared to combined surgery and adjuvant RT, a factor particularly critical for tumors of the midface and periorbital regions. In a cohort of 84 patients, no statistically significant differences were observed in 5‑year OS (51% for both groups) or DFS (40% for RT alone vs. 43% for surgery plus RT) [47]. The rarity of MCC presents significant challenges for patient accrual in prospective trials. This was exemplified by the RATIONAL MCC study, which aimed to compare radical surgery with definitive radiotherapy. The trial was prematurely terminated after failing to meet its feasibility targets for randomization, highlighting the difficulty of conducting large-scale prospective research in this orphan disease [48].

## Systemic therapies and combination with radiotherapy

When interpreting the current literature with mainly retrospective patient cohorts, it is essential to note that the 8th edition of the AJCC/Union for International Cancer Control (UICC) staging system was implemented in 2017—coinciding with the clinical introduction of immunotherapy. These dual shifts in classification and treatment standards must be considered when evaluating long-term outcomes and treatment regimens.

Clinical trials investigating the integration of immunotherapy and RT in MCC are currently ongoing, with results from several landmark studies eagerly awaited. These include the CARTA trial (Clinicaltrials: NCT04792073) assessing avelumab combined with comprehensive ablative RT in metastatic settings and a randomized phase II trial (Clinicaltrials: NCT03304639) comparing pembrolizumab alone versus pembrolizumab plus SBRT. In the adjuvant setting, the STAMP trial (Clinicaltrials: NCT03712605) and the ADAM trial (Clinicaltrials: NCT03271372) are evaluating the benefit of PD-L1 inhibition following definitive surgery and/or radiation, while the neoadjuvant potential of lenvatinib plus pembrolizumab is also under investigation (Clinicaltrials: NCT04869137) [49].

## Conclusion

Adjuvant RT of the tumor bed within 8 weeks after surgery remains the standard of care in MCC. In cases of lymph node involvement, lymph node dissection and/or radiotherapy of the affected nodal basin are recommended. For patients who are not eligible for surgery due to inoperability, refusal of surgery, or significant frailty, definitive local RT can achieve sufficient disease control.

## Data Availability

All data supporting the findings of this work are included in the article.

## References

[1] Hernandez LE, Mohsin N, Yaghi M et al (2022) Merkel cell carcinoma: An updated review of pathogenesis, diagnosis, and treatment options. Dermatologic Therapy. 10.1111/dth.15292 34967084

[2] Lugowska I, Becker JC, Ascierto PA et al (2024) Merkel-cell carcinoma: ESMO–EURACAN Clinical Practice Guideline for diagnosis, treatment and follow-up. ESMO Open 9(5):102977. 10.1016/j.esmoop.2024.102977 38796285 PMC11145756

[3] Becker JC, Stang A, Schrama D et al (2024) Merkel Cell Carcinoma: Integrating Epidemiology, Immunology, and Therapeutic Updates. Am J Clin Dermatol 25(4):541–557. 10.1007/s40257-024-00858-z 38649621 PMC11193695

[4] Stang A, Becker JC, Nghiem P et al (2018) The association between geographic location and incidence of Merkel cell carcinoma in comparison to melanoma: An international assessment. Eur J Cancer 94:47–60. 10.1016/j.ejca.2018.02.003 29533867 PMC6019703

[5] Olsen CM, Pandeya N, Whiteman DC (2021) International Increases in Merkel Cell Carcinoma Incidence Rates between 1997 and 2016. J Invest Dermatol 141(11):2596–2601.e1. 10.1016/j.jid.2021.04.007 33932460

[6] Stang A, Möller L, Wellmann I et al (2024) Incidence and Relative Survival of Patients with Merkel Cell Carcinoma in North Rhine-Westphalia, Germany, 2008–2021. Cancers 16(11):2158. 10.3390/cancers16112158 38893275 PMC11171844

[7] Gonzalez MR, Bryce-Alberti M, Portmann-Baracco A et al (2022) Treatment and survival outcomes in metastatic Merkel cell carcinoma: Analysis of 2010 patients from the SEER database. Cancer Treat Res Commun 33:100665. 10.1016/j.ctarc.2022.100665 36446191

[8] Silling S, Kreuter A, Gambichler T et al (2022) Epidemiology of Merkel Cell Polyomavirus Infection and Merkel Cell Carcinoma. Cancers 14(24):6176. 10.3390/cancers14246176 36551657 PMC9776808

[9] Becker JC, Stang A, DeCaprio JA et al (2017) Merkel cell carcinoma. Nat Rev Dis Primers. 10.1038/nrdp.2017.77 29072302 PMC6054450

[10] Eich HT, Eich D, Staar S et al (2002) Role of postoperative radiotherapy in the management of Merkel cell carcinoma. American journal of clinical oncology 25(1):50–56. 10.1097/00000421-200202000-00011 11823697

[11] Lodde GC, Leiter U, Gesierich A et al (2025) Clinical course of Merkel cell carcinoma: A DeCOG multicenter study of 1049 patients. Eur J Cancer 221:115406. 10.1016/j.ejca.2025.115406 40228429

[12] Hanlon A (2019) Merkel Cell Carcinoma. JAMA Dermatol 155(9):1096–1096. 10.1001/jamadermatol.2019.2068 31365026

[13] Becker JC, Beer AJ, DeTemple VK et al (2023) S2k-Leitlinie - Merkelzellkarzinom - Update 2022. J Dtsch Dermatol Ges 21(3):305–317. 10.1111/ddg.14930_g 36929546

[14] Dai YH, Wang YF, Shen PC et al (2021) Radiosensitivity index emerges as a potential biomarker for combined radiotherapy and immunotherapy. NPJ Genom Med 6(1):40. 10.1038/s41525-021-00200-0 34078917 PMC8172905

[15] Choucair K, Morand S, Stanbery L et al (2020) TMB: a promising immune-response biomarker, and potential spearhead in advancing targeted therapy trials. Cancer gene therapy 27(12):841–853. 10.1038/s41417-020-0174-y 32341410

[16] Passerini S, Fracella M, Ferlosio A et al (2025) Investigation of mRNA expression levels of DNA damage response genes in Merkel Cell Polyomavirus-positive Merkel Cell Carcinoma: a pilot study. Discov Oncol 16(1):852. 10.1007/s12672-025-02651-8 40399437 PMC12095753

[17] Pedersen EA, Verhaegen ME, Joseph MK et al (2024) Merkel cell carcinoma: updates in tumor biology, emerging therapies, and preclinical models. Front Oncol 14:1413793. 10.3389/fonc.2024.1413793 39136002 PMC11317257

[18] Moore SA, Narayanan D, Simonette RA et al (2022) Selinexor is a novel inhibitor of DNA damage response in Merkel cell carcinoma. Clin Exp Dermatol 47(7):1354–1357. 10.1111/ced.15120 35120268

[19] Foote M, Harvey J, Porceddu S et al (2010) Effect of radiotherapy dose and volume on relapse in Merkel cell cancer of the skin. Adv Radiat Oncol 77(3):677–684. 10.1016/j.ijrobp.2009.05.067 19906498

[20] Alexander NA, Schaub SK, Goff PH et al (2024) Increased risk of recurrence and disease-specific death following delayed postoperative radiation for Merkel cell carcinoma. J Am Acad Dermatol 90(2):261–268. 10.1016/j.jaad.2023.07.1047 37778663 PMC11260506

[21] Ma KL, Sharon CE, Tortorello GN et al (2023) Delayed time to radiation and overall survival in Merkel cell carcinoma. J Surg Oncol 128(8):1385–1393. 10.1002/jso.27421 37622232 PMC11338135

[22] Bhatia S, Storer BE, Iyer JG et al (2016) Adjuvant Radiation Therapy and Chemotherapy in Merkel Cell Carcinoma: Survival Analyses of 6908 Cases From the National Cancer Data Base. JNCI Cancer Spectr 108(9):djw042. 10.1093/jnci/djw042 27245173 PMC6059142

[23] Petrelli F, Ghidini A, Torchio M et al (2019) Adjuvant radiotherapy for Merkel cell carcinoma: A systematic review and meta-analysis. Clin Transl Radiat Oncol 134:211–219. 10.1016/j.radonc.2019.02.015 31005218

[24] Wong WG, Stahl K, Olecki EJ et al (2021) Survival Benefit of Guideline-Concordant Postoperative Radiation for Local Merkel Cell Carcinoma. Journal of Surgical Research 266:168–179. 10.1016/j.jss.2021.03.062 34015514

[25] Patel SA, Qureshi MM, Sahni D et al (2017) Identifying an Optimal Adjuvant Radiotherapy Dose for Extremity and Trunk Merkel Cell Carcinoma Following Resection. JAMA Dermatol 153(10):1007. 10.1001/jamadermatol.2017.2176 28746702 PMC5710335

[26] Karki S, Kersch CN, Melson RA et al (2026) No Association Between Radiation Dose and Clinical Outcomes in Merkel Cell Carcinoma in the Veteran Population. Adv Radiat Oncol 11(6):102029. 10.1016/j.adro.2026.102029 42017016 PMC13092482

[27] Sims JR, Grotz TE, Pockaj BA et al (2018) Sentinel lymph node biopsy in Merkel cell carcinoma: The Mayo Clinic experience of 150 patients. Surg Oncol 27(1):11–17. 10.1016/j.suronc.2017.10.005 29549898

[28] Straker RJ, Carr MJ, Sinnamon AJ et al (2021) Predictors of False Negative Sentinel Lymph Node Biopsy in Clinically Localized Merkel Cell Carcinoma. Ann Surg Oncol 28(12):6995–7003. 10.1245/s10434-021-10031-z 33890195

[29] Jouary T, Leyral C, Dreno B et al (2012) Adjuvant prophylactic regional radiotherapy versus observation in stage I Merkel cell carcinoma: a multicentric prospective randomized study. Facts Views Vis Obgyn 23(4):1074–1080. 10.1093/annonc/mdr318 21750118

[30] Kang SH, Haydu LE, Goh RYH et al (2012) Radiotherapy is associated with significant improvement in local and regional control in Merkel cell carcinoma. Radiat Oncol. 10.1186/1748-717X-7-171 23075308 PMC3494567

[31] Lee JS, Durham AB, Bichakjian CK et al (2019) Completion Lymph Node Dissection or Radiation Therapy for Sentinel Node Metastasis in Merkel Cell Carcinoma. Ann Surg Oncol 26(2):387–394. 10.1245/s10434-018-7072-7 30556118

[32] Cramer JD, Suresh K, Sridharan S (2020) Completion lymph node dissection for merkel cell carcinoma. Am J Surg 220(4):982–986. 10.1016/j.amjsurg.2020.02.018 32087988

[33] Kim S, Wuthrick E, Blakaj D et al (2022) Combined nivolumab and ipilimumab with or without stereotactic body radiation therapy for advanced Merkel cell carcinoma: a randomised, open label, phase 2 trial. Lancet (London, England) 400(10357):1008–1019. 10.1016/S0140-6736(22)01659-2 36108657 PMC9533323

[34] Iyer JG, Parvathaneni U, Gooley T et al (2015) Single-fraction radiation therapy in patients with metastatic Merkel cell carcinoma. Cancer Med 4(8):1161–1170. 10.1002/cam4.458 25908228 PMC4559027

[35] Liu KX, Milligan MG, Schoenfeld JD et al (2022) Characterization of clinical outcomes after shorter course hypofractionated and standard-course radiotherapy for stage I-III curatively-treated Merkel cell carcinoma. Clin Transl Radiat Oncol 173:32–40. 10.1016/j.radonc.2022.05.012 35595174

[36] Gonzalez L, Rubens M, Yarlagadda S et al (2024) Hypofractionated versus standard fractionation radiotherapy for merkel cell carcinoma. Radiat Oncol. 10.1186/s13014-024-02516-4 39394124 PMC11468079

[37] Cook MM, Schaub SK, Goff PH et al (2020) Postoperative, Single-Fraction Radiation Therapy in Merkel Cell Carcinoma of the Head and Neck. Adv Radiat Oncol 5(6):1248–1254. 10.1016/j.adro.2020.07.003 32838069 PMC7373007

[38] Dykstra M, Parra CE, Hayman JA et al (2025) Adjuvant single-fraction radiotherapy to the resected primary tumor site and stage IIIA regional disease results in high locoregional control in Merkel cell carcinoma in a single-institution retrospective study. J Am Acad Dermatol 92(6):1438–1439. 10.1016/j.jaad.2025.01.099 39987990

[39] Cimbak N, Barker CA (2016) Short-Course Radiation Therapy for Merkel Cell Carcinoma: Relative Effectiveness in a “Radiosensitive” Tumor. Adv Radiat Oncol 96(2):S160. 10.1016/j.ijrobp.2016.06.387

[40] Colgan MB, Tarantola TI, Weaver AL et al (2012) The predictive value of imaging studies in evaluating regional lymph node involvement in Merkel cell carcinoma. J Am Acad Dermatol 67(6):1250–1256. 10.1016/j.jaad.2012.03.018 22552001

[41] Concannon R, Larcos GS, Veness M (2010) The impact of 18F-FDG PET-CT scanning for staging and management of Merkel cell carcinoma: Results from Westmead Hospital, Sydney, Australia. J Am Acad Dermatol 62(1):76–84. 10.1016/j.jaad.2009.06.021 20082888

[42] Poulsen M, Macfarlane D, Veness M et al (2018) Prospective analysis of the utility of 18- FDG PET in Merkel cell carcinoma of the skin: A Trans Tasman Radiation Oncology Group Study, TROG 09:03. J Med Imaging Radiat Oncol 62(3):412–419. 10.1111/1754-9485.12705 29405630

[43] Byrne K, Siva S, Chait L et al (2015) 15-Year Experience of 18 F-FDG PET Imaging in Response Assessment and Restaging After Definitive Treatment of Merkel Cell Carcinoma. J Nucl Med 56(9):1328–1333. 10.2967/jnumed.115.158261 26159592

[44] Gunaratne DA, Howle JR, Veness MJ (2017) Definitive radiotherapy for Merkel cell carcinoma confers clinically meaningful in-field locoregional control: A review and analysis of the literature. J Am Acad Dermatol 77(1):142–148.e1. 10.1016/j.jaad.2017.02.015 28495499

[45] Mortier L (2003) Radiotherapy Alone for Primary Merkel Cell Carcinoma. Arch Dermatol 139(12):1587. 10.1001/archderm.139.12.1587 14676075

[46] Pape E, Rezvoy N, Penel N et al (2011) Radiotherapy alone for Merkel cell carcinoma: A comparative and retrospective study of 25 patients. J Am Acad Dermatol 65(5):983–990. 10.1016/j.jaad.2010.07.043 21641081

[47] Dubois M, Abi Rached H, Escande A et al (2021) Outcome of early stage Merkel carcinoma treated by exclusive radiation: a study of 53 patients. Radiat Oncol. 10.1186/s13014-021-01815-4 33990201 PMC8120723

[48] Steven NM, Pirrie S, Jefferson-Hulme Y et al (2025) Rational treatment selection for primary Merkel Cell Carcinoma: a Rational MCC RCT comparing surgery and radiotherapy with parallel observational study. Efficacy Mech Eval. 10.3310/EEWD6684 41370622

[49] Tai P, Alqaisi O, Al-Ghabeesh S et al (2025) Immune Checkpoint Inhibitors in Merkel Cell Carcinoma of the Skin: A 2025 Comprehensive Review. Cancers 17(19):3272. 10.3390/cancers17193272 41097798 PMC12523286

